# Salinity Alters Toxicity of Commonly Used Pesticides in a Model Euryhaline Fish Species (*Menidia beryllina*)

**DOI:** 10.3390/toxics9050114

**Published:** 2021-05-20

**Authors:** Sara J. Hutton, Scott J. St. Romain, Emily I. Pedersen, Samreen Siddiqui, Patrick E. Chappell, J. Wilson White, Kevin L. Armbrust, Susanne M. Brander

**Affiliations:** 1 Department of Environmental and Molecular Toxicology, Oregon State University, Corvallis, OR 97331, USA; huttonsa@oregonstate.edu; 2 Department of Environmental Sciences, Louisiana State University, Baton Rouge, LA 70803, USA; sstrom6@lsu.edu (S.J.S.R.); armbrust@lsu.edu (K.L.A.); 3 Department of Fisheries, Wildlife, and Conservation Sciences, Coastal Oregon Marine Experiment Station, Oregon State University, Newport, OR 97365, USA; pederemi@oregonstate.edu (E.I.P.); samreen.siddiqui@oregonstate.edu (S.S.); will.white@oregonstate.edu (J.W.W.); 4 Department of Biomedical Sciences, Oregon State University, Corvallis, OR 97331, USA; patrick.chappell@oregonstate.edu

**Keywords:** LC_10_, LC_50_, ecotoxicology, global climate change, salinity regimes, multiple stressors, marine toxicity

## Abstract

Changing salinity in estuaries due to sea level rise and altered rainfall patterns, as a result of climate change, has the potential to influence the interactions of aquatic pollutants as well as to alter their toxicity. From a chemical property point of view, ionic concentration can increase the octanol–water partition coefficient and thus decrease the water solubility of a compound. Biologically, organism physiology and enzyme metabolism are also altered at different salinities with implications for drug metabolism and toxic effects. This highlights the need to understand the influence of salinity on pesticide toxicity when assessing risk to estuarine and marine fishes, particularly considering that climate change is predicted to alter salinity regimes globally and many risk assessments and regulatory decisions are made using freshwater studies. Therefore, we exposed the Inland Silverside (*Menidia beryllina*) at an early life stage to seven commonly used pesticides at two salinities relevant to estuarine waters (5 PSU and 15 PSU). Triadimefon was the only compound to show a statistically significant increase in toxicity at the 15 PSU LC_50_. However, all compounds showed a decrease in LC_50_ values at the higher salinity, and all but one showed a decrease in the LC_10_ value. Many organisms rely on estuaries as nurseries and increased toxicity at higher salinities may mean that organisms in critical life stages of development are at risk of experiencing adverse, toxic effects. The differences in toxicity demonstrated here have important implications for organisms living within estuarine and marine ecosystems in the Anthropocene as climate change alters estuarine salinity regimes globally.

## 1. Introduction

Estuaries are incredibly diverse and productive ecosystems that provide many ecosystem services and essential habitat to diverse species [1]. A number of organisms spend their entire life histories in estuaries; some rely on estuarine habitat exclusively for reproduction and as a nursery in early life stages, while others pass through only during migration [2]; for these reasons, it is important to understand the toxicological effects of different contaminants on estuarine and marine organisms. However, despite the importance of estuaries, exposure and toxicity data for many pesticides is lacking in many estuarine and marine species [3], complicating the ability to conduct adequate risk assessments. Furthermore, as climate change worsens, water quality parameters in estuaries (e.g., salinity) are becoming more variable as sea level rises and rainfall patterns change [4]. Sea level rise is already causing salinity intrusion in estuaries and bay regions [5], reducing habitat and potentially exposing organisms residing in lower saline niches to higher salinities. Changes in rainfall patterns will likely cause an increase in storm events and stormwater runoff, resulting in more frequent pulses of contaminants into estuaries, whereas increased occurrence of drought has already been linked to increased salinity and shifts in organism’s abundance and predation [6,7,8]. These abiotic changes in estuaries are not abstract or a concern for the future, as examples of these challenges already exist. For example, the Delta Smelt (*Hypomesus transpacificus*) is listed as critically endangered and spawns in waters close to 0.5 PSU where the larvae stay before migrating to slightly higher salinities (1–6 PSU) [9]. Changes in rainfall patterns and increased salinity can alter salinity regimes and place stress on the developing larvae, especially when exposed to pesticide pollution [10]. As organisms experience additional stress from their environment due to climate change, their ability to tolerate pollution can be compromised [11,12]. Changes in water quality parameters and increased frequency of stormwater events make additional ecotoxicology studies in estuaries all the more important.

Fungicides, herbicides, and insecticides (further referred to collectively as pesticides) enter the aquatic environment due to their frequent use in both agriculture and household products, which results in runoff and spray drift [13,14]. Once they enter the watershed, pesticides travel throughout the aquatic ecosystem, where they can be ultimately deposited in estuarine and marine environments. Many pesticides are used in agricultural, residential, and industrial (non-agricultural) applications within close proximity to estuaries and coastal regions due to the larger number of crops and high human population along coastlines, further increasing pesticide pollution in these ecosystems [15,16]. Estuaries can serve as a sink for many pollutants that enter through stormwater runoff, wastewater discharge, and pesticide drift, where they are frequently detected in surface water [16,17].

As discussed, estuarine salinity is likely to be heavily influenced by climate change [12]. In regard to pesticides in estuaries, studies have demonstrated that salinity can alter their chemical properties, which can cause toxicity [18]. As salinity increases, the aqueous solubility of an organic chemical constituent typically decreases (salting-out effect), increasing the potential for bioaccumulation [19]. In a study by Saranjampour et al., it was found that all compounds tested showed an increased log K_OW_ and decreased water solubility in saline compared to fresh water [18]. While increased bioaccumulation can be a result of abiotic changes, organism physiology can also play a role in the increased uptake of compounds in higher saline environments. For example, the Pacific oyster (*Crassostrea gigas*) bioaccumulated more perfluorinated compounds at higher salinities (10–34 PSU), which was attributed to changes in both organism physiology (increased dietary uptake) as well as changes in chemical properties [20]. Other studies have shown potential interactions of pesticides and salinity. Inland Silverside (*Menidia beryllina)* exposed as juveniles to waters collected from the San Francisco Bay, which is contaminated with anthropogenic waste, including pesticides, showed decreased growth and altered gene expression in water adjusted to 20 PSU with a 10 °C increase in temperature compared to those at 10 PSU [21]. Mummichog (*Fundulus heteroclitus)* exposed to atrazine under varying salinities (3, 15, and 35 PSU) were unable to maintain osmotic balance at non-isosmotic salinities (3 and 35 PSU) in the absence of atrazine. Mummichog is typically found in the water around 10–12 PSU, but exposure to atrazine impacted their ability to tolerate a range of salinities [22]. The same species also showed non-monotonic bioaccumulation of 17-β-ethynylestradiol, with more uptake at 16 PSU than 0 PSU or 32 PSU. Altered gill morphology at the different salinities was correlated with differences in bioaccumulation, since the gills are the route of exposure for many contaminants [23]. Overall, the interaction of salinity and contaminants on toxicity is likely complicated [10,23,24].

In this study, we conducted range finding and constructed dose–response curves for seven common pesticides (myclobutanil, chlorpyrifos, bifenthrin, dicloran, paraquat, penconazole, and triadimefon) at two salinities relevant to estuaries, 5 PSU and 15 PSU. Bifenthrin is a type 1 pyrethroid that disrupts endocrine function and lowers the reproductive output of Inland Silverside (*Menidia beryllina*) [25]. It is a heavily used insecticide found globally in estuarine surface waters and sediments [15]. Chlorpyrifos is an organophosphate pesticide that disrupts acetylcholinesterase activity in the brain [26]. Chlorpyrifos was used widely within the Chesapeake Bay watershed [27], and despite recent bans, is still used globally [28]. Dicloran is a fungicide used to prevent crop fungal diseases in the Western and Southern United States; it is applied through aerial spray, often in areas close to estuaries [29,30]. Myclobutanil is a fungicide typically applied throughout the United States and is used on a number of agricultural crops, such as vegetables and grapes, and causes altered gene expression and toxicity to the liver [31,32]. Paraquat is an herbicide, used heavily around the world and found within estuarine sediments and surface waters globally [33,34]. It is a hepatotoxicant, with a reported environmental half-live of around two years, making it extremely persistent in the environment [35]. Penconazole and triadimefon are azole fungicides that cause cardiotoxicity across taxa and are found in surface and groundwater through runoff and spray drift [36,37].

To understand the impact salinity has on toxicity, we exposed the model estuarine fish, Inland Silverside (*Menidia beryllina*), to all seven pesticides, individually, at two salinities relevant to estuaries (5 and 15 PSU) and at which they are found in the wild [38]. Inland Silversides are a common EPA Whole Effluent Toxicity test species used in the regulation of effluents discharged into estuarine and marine waterways. They are native to the Eastern and Gulf coasts of the United States and non-native (introduced) to the California coast [38,39]. They are moderately sensitive to contaminants and can tolerate a wide range of salinities (0–35 PSU), making them an ideal model fish species for studying the toxicity of contaminants relevant to estuaries [38]. Based on previous studies showing that compounds with a higher log K_OW_ increased their affinity for the solid phase as salinity increased [40], we hypothesized that our study pesticides with higher log K_OW_ values would show the most significant difference in toxicity. Therefore, this study aimed to determine the extent to which toxicity changed under different exposure salinities (5 PSU and 15 PSU).

## 2. Materials and Methods

### 2.1. Exposure Chemicals

For the exposure assays, bifenthrin (98% purity, CAS: 82657-04-3, part no. N-11203-100MG), chlorpyrifos (99.5% purity, CAS: 2921-88-2, part no. N-11459-250MG), dicloran (99.4% purity, CAS 99-30-9, part no. N-11678-250MG), myclobutanil (99.5% purity, CAS: 88671-89-0, part no. N-13261-100MG), paraquat dichloride hydrate (99.5% purity, CAS: 75365-73-0, part no. N-12818-500MG), penconazole (99.5% purity, CAS: 66246-88-6, part no. N-12822-100MG), and triadimefon (99.5% purity, CAS: 43121-43-3, part no. N-13636-500MG) were obtained from Chem Service (West Chester, PA, USA). Paraquat stock solution was prepared in deionized water and all other compounds were prepared in HPLC-grade methanol from Fisher Scientific (Waltham, MA, USA). All stock solutions were prepared by serial dilution, with a final solvent concentration of 0.1%.

### 2.2. Organism Husbandry

Brood stock adult Inland Silversides were housed at the Oregon State University Hatfield Marine Science Center under Animal Care and Use Program (ACUP) protocol #4999. Adult fish were held at 23 °C, 19–21 PSU, and fed a combination of Hikari tropical micro pellets (Kyorin Food Industries Ltd., Kasai City, Japan), Hikari freeze-dried tubifex worms (Kyorin Food Industries Ltd.), Hikari frozen mysid shrimp (Kyorin Food Industries Ltd., Kasai City, Japan), and live *Artemia nauplii* hatched from Brine Shrimp Eggs (Brine Shrimp Direct, Ogden, UT, USA) supplemented with Selcon™ (American Marine Inc., Ridgefield, CT, USA). Brood fish were approximately 1.5–2 years old at the time of spawning. Spawning was induced by adding spawning substrate (wool, unbleached yarn) to brood stock tanks and lowering the salinity by 2–3 PSU by adding reverse osmosis freshwater. Spawning substrate was left in the tanks for 16–18 h after which the substrate was removed and transported to the Oregon State University main campus.

### 2.3. Bioassays

Inland Silverside embryos were removed from the spawning substrate, assessed for fertilization with a VWR VistaVision Dissecting Scope (VWR International, Radnor, PA, USA), and randomly placed in exposure wells with 1 mL of clean artificial seawater (ASW) made from reverse osmosis water and Instant Ocean Sea Salt (Spectrum Brands, Blacksburg, VA, USA). Once all well plates (Supplemental Material and Video S1) were loaded, each test was initiated by pouring the appropriate exposure solution into the well plate until embryos were submerged in 8 mL of exposure solution. Organisms were exposed from <24 h post fertilization (hpf) as embryos to 96 h post hatch (hph) larvae for a total exposure period of 12 days. The average hatch date for the control organisms was 7.92 ± 0.43, with the median hatching on day 8. There were no significant differences in the control organism’s hatching rates due to salinity (*p* value ≥ 0.05, one-way ANOVA). After hatching, fish larvae, which still had their yolk sacs, were fed Gemma Microdiet (Skretting, Westbrook, ME, USA) once per day and were allowed to feed for at least two hours prior to water changes. The tests were performed in a temperature-controlled room maintained at an ambient temperature of 24 ± 1 °C on a 14:10 light–dark cycle at a light intensity of 224–256 lux (measured with Lux Light Meter Pro version 2.1.1 developed by Marina Polyanskaya on iPhone X, Apple Inc. Cupertino, CA, USA). Temperature, pH, salinity, and dissolved oxygen were measured daily before and after water changes using a YSI Professional Plus Quatro water quality meter (YSI Incorporated, Yellow Springs, OH, USA) and API Ammonia Test Kit and can be found in Table S1.

Range finding exposures were conducted at two separate salinities (5 PSU and 15 PSU) for bifenthrin, chlorpyrifos, dicloran, myclobutanil, paraquat, penconazole, and triadimefon. This suite of chosen contaminants represents commonly used pesticides with a range of log K_OW_ values, water solubilities, and target organs [41]. Six concentrations (Table 1), including a solvent control, were assessed per test to determine the appropriate dose–response relationships. Initial test concentrations were determined from existing mortality data from the United State EPA ECOTOX database. Where testing on saltwater species was not available, freshwater data were used to supplement. Penconazole does not have mortality data available for fish on ECOTOX and tebuconazole was used to estimate its freshwater toxicity instead. The fourth concentration was selected based off existing mortality data and increased by one magnitude to the fifth concentration to ensure mortality. All lower concentrations were decreased by one subsequent magnitude to ensure an appropriate dose response could be achieved. Freshwater LC_50_ values compared in the results are expressed as a range of the minimum and maximum reported LC_50_ values in the ECOTOX database for Rainbow Trout (*Oncorhynchus mykiss*) 96-h acute tests.

Chemical stock solutions were made at the start of each test from which new exposure solutions were made daily in ASW immediately prior to water changes. There were six replicates per concentration and two organisms per replicate. A 75% water change was performed every 24 h at which time debris and dead fish were removed and survival were assessed from each beaker. At the end of the exposure period, the final survival data were recorded, and larval fish were humanely sacrificed. Exposures were approved and conducted under the Oregon State University Institutional Animal Care and Use Committee (IACUC) protocol #0035.

### 2.4. Analytical Chemistry

Two 500 mL exposure solutions were collected from the middle concentrations of chlorpyrifos (acidified to <pH 7 to prevent hydrolysis), dicloran, myclobutanil, paraquat, penconazole, and triadimefon and three 100 mL samples were collected from bifenthrin at 5 PSU to confirm the chemical dosing methodology. The third concentration was selected for analysis since it reflected the range where we hypothesized LC_50_ values would fall. Samples were shipped to Louisiana State University, Baton Rouge, LA, for analysis.

For analytical chemistry, bifenthrin (98% purity, CAS: 82657-04-3, part no. N-11203-100MG), chlorpyrifos (99.5% purity, CAS: 2921-88-2, part no. N-11459-250MG), myclobutanil (99.5% purity, CAS: 88671-89-0, part no. N-13261-100MG), paraquat dichloride hydrate (99.5% purity, CAS: 75365-73-0, part no. N-12818-500MG), penconazole (99.5% purity, CAS: 66246-88-6, part no. N-12822-100MG), and triadimefon (99.5% purity, CAS: 43121-43-3, part no. N-13636-500MG) were obtained from Chem Service (West Chester, PA, USA), and dicloran (96% purity, CAS: 99-30-9, part no. D67820-5G) was obtained from Sigma Aldrich (St. Louis, MO, USA). Solvents used include methanol, acetonitrile, water, and dichloromethane (chromatographic grade, VWR). Formic acid was 50% in water from Fluka Analytical (St. Gallen, Switzerland) and ammonium formate (97%) was from Fisher Scientific. All solvents and reagents used were analytical grade and were used without further purification.

#### 2.4.1. Extraction and Sample Preparation

The bifenthrin and chlorpyrifos residues were extracted from water samples using C8 solid-phase extraction (SPE) cartridges (Thermo Fisher, 500 mg sorbent, 3 mL reservoir volume) placed on a vacuum manifold to capture the analytes. Cartridges were preconditioned with 3 mL of methanol and 3 mL of deionized water prior to adding the sample. The residual pesticide was eluted from the cartridge with 1.5 mL of dichloromethane for both compounds. All other compounds did not require solid-phase extraction and were analyzed directly by HPLC. Recoveries averaged 80% ± 2.28% (*n* = 6) and 76% ± 0.56% (*n* = 5) for bifenthrin and chlorpyrifos.

#### 2.4.2. High Performance Liquid Chromatographic (HPLC) Analysis

An Agilent 1260 Infinity high performance liquid chromatography (Santa Clara, CA, USA) coupled with a diode array detector was used to directly measure dicloran, myclobutanil, paraquat, penconazole, and triadimefon in water samples. Dicloran, myclobutanil, and triadimefon were separated on an Agilent Zorbax Eclipse C8 (Santa Clara, CA, USA) column using a gradient mobile phase consisting of acetonitrile and water with a flow rate of 0.7 mL/min and injection volume of 40 μL. Penconazole was analyzed under the same conditions but was separated using a Zorbax Eclipse C18 column. Dicloran, myclobutanil, penconazole, and triadimefon were detected at 380 nm, 230 nm, 220 nm, and 225 nm, respectively. Paraquat was separated using an Agilent Infinity Lab Poroshell 120 HILIC-Z column (Santa Clara, CA, USA) using a gradient mobile phase consisting of 0.05 M ammonium formate in water (pH: 3) and 0.1% formic acid in acetonitrile with a 0.5 mL min^−1^ flow rate and 2.5 μL injection volume with detection at 258 nm.

#### 2.4.3. Gas Chromatographic Analysis

A Bruker Scion TQ 456-GC/MS/MS (Billerica, MA, USA) was used to measure bifenthrin and chlorpyrifos in SPE extracts. Residues were separated on a Restek RXI-PAH column (Bellefonte, PA, USA, 60 m length, 0.25 mm internal diameter, and 0.10 micron film thickness) programmed at 90 °C for 3 min and then ramped up 5 °C/min to 300 °C where the temperature was held for 10 min. The carrier gas was ultra-purity helium, and the collision gas was ultra-purity argon. External standards were used to quantify all the concentrations of all analytes, whether measured by GC or HPLC.

### 2.5. Statistical Analysis

Statistical analysis was conducted in R version 4.0 (Vienna, Austria) and R Studio version 1.3.1093 (Boston, MA, US). The dose–response analysis consisted of fitting two-parameter log-logistic models to all seven compounds at both salinities (14 fits in total) using maximum likelihood optimization in the package *drc* version 3.0-1 [42]. Goodness-of-fit was quantified using Nagelkerken’s (1991) pseudo-R^2^ formula, which can be found in Table S2 [43]. All data generated in this study and R code used for statistical analysis has been made publicly available and can be found at https://doi.org/10.5281/zenodo.4735670. To determine if the detected differences at 10 and 50% lethal concentration (LC_10_ and LC_50_ values) between the salinities were statistically significant, a Monte Carlo randomized resampling simulation was performed with 5000 simulations for each dose–response analysis. In the Monte Carlo procedure, the salinity treatments for all of the replicates in the dataset were permuted (keeping the sample sizes for each salinity the same), then new dose–response curves were fit to each salinity treatment. The *p*-value testing whether the observed difference in LC_10_ and LC_50_ values was greater than that expected by random chance was calculated as the proportion of Monte Carlo simulations in which the difference in LC values was greater than the original observed difference [44]. A *p*-value ≤ 0.05 was considered statistically significant. All values are reported as the mean ± standard error. All statistical analysis and dose–response curves were made and reported with nominal concentrations.

## 3. Results and Discussion

Analytical results can be found in Table 2. All values are reported as nominal. The bifenthrin and chlorpyrifos samples were measured at 58% and 56.4%, respectively. All other concentrations were close to nominal. The lower detected bifenthrin and chlorpyrifos concentrations are likely in part due to the lower recovery rates for these compounds (80% ± 2.28% (*n* = 6) and 76% ± 0.56% (*n* = 5) for bifenthrin and chlorpyrifos, respectively) as well as the shorter half-life of chlorpyrifos [45].

**Figure 1:**
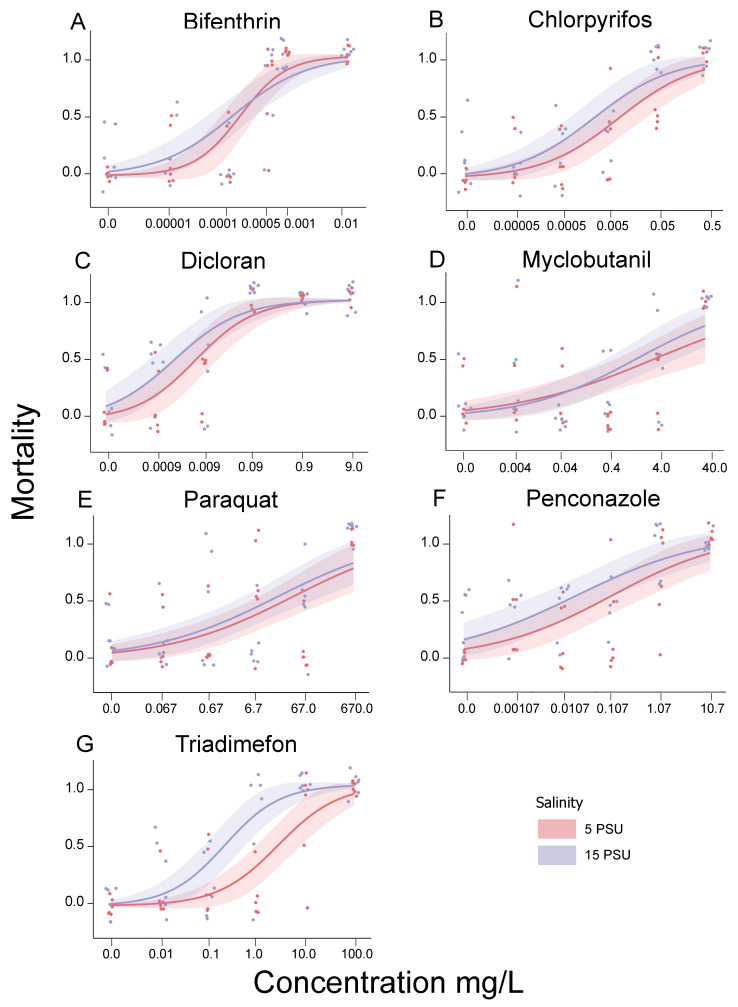
Dose response curves for (**A**) bifenthrin, (**B**) chlorpyrifos, (**C**) dicloran, (**D**) myclobutanil, (**E**) paraquat, (**F**) penconazole, and (**G**) triadimefon at 5PSU and 15PSU. Triadimefon had a statistically significant difference in toxicity between the two salinities with higher toxicity at the LC50 value in the 15 PSU exposure. Data points are jittered along the x and y axes for visual clarity.

Triadimefon had significantly greater toxicity at the higher salinity, with an LC_50_ an order of magnitude lower at 15 PSU than 5 PSU (*p* = 0.0021, Table 3, Figure 1). For the other compounds, the LC_50_ values also trended lower (more toxic) for organisms exposed at 15 PSU than 5 PSU, though those differences were not statistically significant. LC_10_ values were also lower at 15 PSU, but not statistically different from 5 PSU, for all compounds except myclobutanil, which was slightly higher at 5 PSU (Table 3, Figure 1).

Triadimefon was significantly different between the two exposure salinities by an order of one magnitude (2.74 mg/L and 0.218 mg/L for 5 PSU and 15 PSU, respectively) (Table 3). Notably, all other LC_50_ values were lower at the higher salinities, and all but one LC_10_ was lower, although these were not significantly different. The detected LC_50_ values for bifenthrin, chlorpyrifos, and paraquat fell within the range of those reported for Rainbow Trout 96-h acute toxicity data (Table 3). Myclobutanil’s LC_50_ values fell within the same magnitude but where slightly lower than those reported for Rainbow Trout (Table 3). Triadimefon’s LC_50_ value for 5 PSU was also within the same magnitude of the lower range of those reported for Rainbow Trout but was a magnitude lower than the higher range (Table 3). Our detected LC_50_ values for dicloran are two magnitudes lower than the reported values for Rainbow Trout although no significant differences were detected between the two salinities.

We did not detect differences in LC values that correlated with a higher log K_OW_ like originally hypothesized, indicating that the reason for the potential differences in toxicity across salinities may not be related solely to changes in chemical properties but also related to mechanisms of toxicity and organism physiology. Shukla et al. found that polycyclic aromatic hydrocarbons with higher log K_OW_ values bioaccumulated less in Tilapia (*Tilapia mossambica*) when exposed to 15 PSU and 30 PSU compared to those in freshwater [46]. A study found that Inland Silverside’s fed permethrin-dosed *Hyalella azteca* at 6 PSU, 13 PSU, and 20 PSU bioaccumulated significantly more permethrin in the lowest salinity [47]. Interestingly, they also found that there were no significant differences in metabolism of permethrin at the difference salinities [47]. Other studies have found that enzyme kinetics may alter toxicity under different salinities. Rainbow Trout (*Oncorhynchus mykiss)* acclimated to 0 and 1.7 PSU, which resulted in changes in metabolic enzyme kinetics and distinctly different biotransformation pathways for fenoxon [48]. Others have found potential differences among mechanisms of toxicity when Delta smelt (*Hypomesus transpacificus*) exposed to bifenthrin and permethrin (log K_OW_ = 6.4 and 6.9, respectfully) showed greater alteration in swimming behavior when exposed to bifenthrin, which is known to have a different mechanism of action than permethrin, which suggested the difference in toxicity maybe due to the compound’s different mechanisms of action and not their physiochemical properties [10,25]. Organism-specific differences may also play a role in whether or not toxicity is seen; in a study comparing two strains of trout, Rainbow and Steelhead Trout (*Oncorhynchus mykiss)* were exposed to 0.1 µg/L and 1.5 µg/L bifenthrin at 0, 8, and 17 PSU, after which Rainbow Trout had significant increases in mortality at 0 PSU in the highest concentration and no significant impacts to mortality for either species at the higher salinities [49]. It was also found that Na+/K+ ATPase α1a expression in the gills was significantly increased in Rainbow Trout at the 17 PSU salinity but not in Steelhead [49]. Additionally, Pawar et al. found that the LC_50_ values increased (decreasing toxicity) with increasing salinity in the White Leg shrimp; our results did not find compounds that had higher LC_50_ values in the 15 PSU exposures, further supporting species-specific differences in toxic response across a salinity gradient [3].

The compounds studied here all have markedly different mechanisms of toxicity. Triadimefon, which showed significant changes in toxicity between the two salinities, causes pericardial edema, elongated hearts, and downregulation of genes involved in regulating ATPase, calcium channel, and cardiac troponin C, which led to increased cardiotoxicity in Zebrafish (*Danio rerio*) [36]. Triadimefon has also been shown to induce hepatotoxicity through CYP enzyme-mediated toxicity in Medaka (*Oryzias latipes*) [32]. While little work has been done on dicloran in fish, it is known to target the liver, spleen, kidneys, and hematopoietic system in rats and has been found to cause toxicity in cardiomyocytes of the Pacific oyster (*Crassostrea virginica)*, likely due to decreased beating rates and cell proliferation [30,50]. Paraquat exposure in the Spotted Snakehead (*Channa punctatus)* had effects on the histology of gills, liver, and kidneys after 24–96 h of exposure [51,52]. Other studies have also found toxicity in the liver of Carp (*Cyprinus carpio*) after paraquat exposure, caused by nitric oxide synthase, decreased antioxidant enzyme response, changes in lipid peroxidation, and inflammatory cytokines [53]. The mechanisms by which hepatotoxicity is induced by triadimefon have been proposed to be related to CYP induction; however, hepatoxicity from dicloran and paraquat are less understood in fish [32]. While both dicloran and triadimefon are known to induce cardiotoxicity, the mechanisms of toxicity appear to be different, which could explain the differences in toxicity seen in this study. The other compounds studied have mechanisms of toxicity and target organs different than triadimefon. Bifenthrin and chlorpyrifos are neurotoxicants where the main target organ is the brain [54,55]. Penconazole, a triazole fungicide that causes toxicity to fungi by inhibiting P450-dependent 14α-demethylase, has been found to mainly affect the gills in fish [56,57]. Myclobutanil bioaccumulates into the gills, viscera, and head of Zebrafish and affect the genes related to cholesterol production. It is known that brackish and marine fish must uptake more water in order to maintain osmoregulation [58], which could lead to increased uptake of pesticides through increased ingestion. While the mechanisms that drive increased toxicity at higher salinities are not yet understood, it is clear that salinity has the potential to alter toxicity. Future work should consider quantifying changes in bioaccumulation at different salinities, as well as measuring enzyme activity and gene regulation related to the known mechanisms of toxicity to better understand how toxic effects may be altered in euryhaline fishes.

A number of our studied pesticides are found within estuaries, highlighting the importance of studying toxicity across a salinity gradient. Bifenthrin is often found in the San Francisco Bay and surrounding watershed up to 9.9 ng/L and has been found in estuaries within China up to 291 ng/L, both of which encompass the LC_10_ values [59,60]. In the USA and globally, chlorpyrifos has been detected within estuaries and surface waters up to 96.0 µg/L, concentrations that encompass the LC_10_ values found in this study [61,62,63]. Myclobutanil has also been detected in global estuarine surface waters at levels close to the LC_10_ values found in this study [64]. Surface water samples collected from the Jiulong River estuary in China contained myclobutanil at concentrations ranging from 0.224 µg/L to 6.73 µg/L, which encompass our findings of LC_10_ values for myclobutanil [65]. The literature on the presence of the other compounds in estuaries is lacking; however, they have been detected in surface waters. Paraquat has been detected at levels up to 0.134 mg/L in global surface waters, which encompasses the LC_10_ values found in this study [66]. The literature on the presence of penconazole in surface waters is scarce, making it difficult to know the environmental relevance of our detected levels of toxicity [67]. Triadimefon has been detected within U.S. surface waters up to 0.2 µg/L, which is lower than the levels of toxicity detected for the LC_50_ and LC_10_ values in this study [68]. The continued presence of pesticides in estuarine surface waters and their potential for change under different salinities emphasizes the need for future ecological risk assessments to consider salinity as a driver for altered toxicity.

Previous literature has demonstrated that a number of the compounds used in this study have notably different chemical behavior in seawater compared to fresh. Bifenthrin has been shown to have an increased log K_OW_ and bioconcentration factor in seawater compared to fresh [18]. However, others have not found significant differences in the conversion of bifenthrin to its metabolites in fresh compared to 17 PSU seawater [49]. This could explain the non-significant differences in toxicity for bifenthrin at our chosen salinities. Salinity and dissolved copper have been correlated with the hydrolysis rate of chlorpyrifos and shorten its half-life by a fifth while speeding up hydrolysis 5-fold [27]. The chlorpyrifos metabolite, chlorpyrifos-oxon, is more toxic than the parent compound. However, despite the potential ecotoxicological implications of this, we did not see increased toxicity of chlorpyrifos in our results. This may be due to the salinities chosen for the current study compared to Liu et al., as we looked at 5 PSU and 15 PSU whereas they studied full-strength seawater compared to freshwater [27]. Similar to our results, the White Leg shrimp (*Litopenaeus vannamei)* also showed little difference in LC_50_ values for chlorpyrifos between 5 PSU and 15 PSU exposures [3]. However, they found a statistically significant decrease in toxicity to chlorpyrifos at 25 PSU compared to 5 PSU. While 25 PSU was outside the range of this current study, future work should look at a larger range of salinities to further understand these differences across a salinity gradient.

An additional novelty of our study is that, to our knowledge, no studies have reported toxicity in marine or estuarine fishes or investigated the impacts of salinity on myclobutanil or penconazole toxicity or chemical behavior. It has been shown that dicloran photodegradation products form at an increased rate in 8 PSU estuarine water compared to distilled freshwater [69]. The toxicological implications of this were demonstrated in Xu et al. when cardiomyocytes from the Pacific oyster (*Crassostrea virginica*) had elevated toxicity after exposure to photolyzed dicloran degradation products compared to standard intermediates [50]. Our study was not conducted under conditions that would create a phototoxic response (i.e., all work was done indoors under artificial lighting), which could explain why we did not detect significant differences in toxicity between the two salinities. Dicloran is considered moderately to highly toxic in freshwater fish; however, to our knowledge, no LC_50_ data exists on dicloran for brackish or marine fish species until now [30].

Paraquat has also been shown to have increased water concentrations in saline water compared to fresh. A study from the Pak Phanang river in Thailand found the sorption rate of paraquat to sediment was slower and the rate of desorption was faster at 10 PSU and 20 PSU compared to freshwater, meaning more paraquat entered the water column at the higher salinities [70]. Paraquat is desorbed from sediments and released into ground and surface waters, likely through interaction with other ions in seawater that compete for binding sites on the sediment and push paraquat into the water column [71]. We did not find significant differences in toxicity between the two salinities for paraquat. However, we did detect a trend towards lower LC_50_ and LC_10_ values that could be explained by the differences in the sorption rate noted above. To the best of our knowledge, no sub-lethal studies of paraquat are available in estuarine or marine fish species and only one study has investigated paraquat-induced mortality in marine species; the marine flathead Gray Mullet (*Mugil cephalus*) was tested along with a hermit crab species (*Paragurus* spp.) and Spiny Dye-Murex *(Murex brandiaris*); the Gray Mullet was found to be the most sensitive (time to lethality) to paraquat exposure [72].

The half-life of triadimefon is almost three times shorter in distilled water vs. seawater (54 vs. 178 min) under irradiation of a 300 W mercury bulb [73]. Triadimefon’s metabolite, triadimenol, has been shown to be less toxic than the parent compound [74]; therefore, under higher saline exposure conditions, triadimefon could induce increased toxicity if metabolism is slowed. These differences in chemical breakdown and metabolism toxicity could account for the difference in toxicity seen in this study. However, as noted above, it is difficult to know exactly what mechanism is attributed to causing the significant difference given the limited literature available on this compound and its effects at increased salinities.

The discrepancies in toxic response between other studies and our findings may be attributed to differences in drug metabolism and physiology between different species. As our study was interested in mortality as an endpoint and exposure at 5 PSU and 15 PSU, it is possible that different responses (for example, more sublethal endpoints) may be detected across different biological scales and different salinity ranges. Studies with more refined ranges may detect more subtle differences in toxicity.

## 4. Conclusions

Overall, our findings show that salinity has the potential to influence toxicity in Inland Silverside, a commonly used estuarine and marine model organism. Our study highlights the need to better understand the influence of salinity on toxicity, especially since the one compound that did have a statistically significant difference in toxicity was not the compound with a higher log K_OW_ (triadimefon’s log K_OW_ is 3.2 compared to bifenthrin with a log K_OW_ of 6.6) [41]. Compared to the 96-h LC_50_ values for Rainbow Trout, our 12-day LC_50_ values fell within or slightly below the freshwater ranges for all compounds except triadimefon. Triadimefon’s 15 PSU LC_50_ was one–two magnitudes lower than the Rainbow Trout range, which correlates with our findings that it was significantly different compared to 5 PSU. Existing literature suggests changes in toxicity across a salinity gradient are related to many factors, including changes in the chemical properties, organism physiology, enzyme kinetics, and metabolic rate of compounds in saltwater, which have the potential to alter toxicity [18,27,48,49]. More work is needed to fully understand the extent to which toxicity changes under different salinities and the mechanisms that drive changes in toxicity. Future work should focus on a range of salinities, including those lower on the gradient, and more refined, sublethal exposures. Our results suggest that tidal cycles may already present a challenge to species living within contaminated estuaries as salinity changes throughout the months and seasons by inducing greater toxicity for some compounds when salinity is higher [75]. Given that salinity regimes as well as other abiotic factors (e.g., temperature and dissolved oxygen levels) in many estuaries are at risk of changing in the future, as a result of climate change, risk assessors and environmental managers should consider future salinity regimes when making decisions related to risk. To adequately protect species living within different salinity regimes, more studies are needed to better understand the mechanisms driving changes in toxicity. Where data is lacking, risk assessments may need to widen their margins of safety to account for potential toxicity that is not fully understood yet.

## Figures and Tables

**Table 1:** Chemicals and concentrations used to conduct range finding and determine the LC_50_ and LC_10_ values at 5 PSU and 15 PSU (* [41]).

Chemical	Conc. 1 mg/L	Conc. 2 mg/L	Conc. 3 mg/L	Conc. 4 mg/L	Conc. 5 mg/L	log K_OW_ *	Water Solubility mg/L *
Bifenthrin	0.00001	0.0001	0.0005	0.001	0.01	6.6	0.1
Chlorpyrifos	0.00005	0.0005	0.005	0.05	0.5	4.7	1.4
Dicloran	0.0009	0.009	0.09	0.9	9.0	2.8	6.3
Myclobutanil	0.004	0.04	0.4	4	40	2.8	132
Paraquat	0.067	0.67	6.7	67	670	−4.5	6.20 × 10^5^
Penconazole	0.00107	0.0107	0.107	1.07	10.7	3.7	73
Triadimefon	0.01	0.1	1	10	100	3.2	64

**Table 2:** Analytical data from the middle concentration of each compound. All concentrations expressed as mg/L (nominal). NA = not available due to sample loss during shipping. All values are reported as the mean ± standard error. Recoveries averaged 80% ± 2.28% (*n* = 6) and 76% ± 0.56% (*n* = 5) for bifenthrin and chlorpyrifos, respectively.

Chemical	Nominal Concentration	Sample 1	Sample 2	Sample 3
Bifenthrin	0.0005	0.00030 ± 0.00000985	0.00030 ± 0.00000465	0.00027 ± 0.00000312
Chlorpyrifos	0.005	0.00282 ± 0.000400	NA	
Dicloran	0.09	0.0830 ± 0.0000676	0.079 ± 0.00014	
Myclobutanil	0.4	0.493 ± 0.00550	0.49 ± 0.0021	
Paraquat	6.7	6.61 ± 0.0150	7.19 ± 0.38	
Penconazole	0.107	0.0966 ± 0.00130	0.112 ± 0.0012	
Triadimefon	1.0	0.966 ± 0.00235	0.896 ± 0.00156	

**Table 3:** LC_50_ and LC_10_ values for all tested compounds at 5 PSU and 15 PSU. All concentrations reported as mg/L (nominal). ^a^ Indicates the difference in LC_50_ between the two salinities was significant, *p* < 0.05. * FW LC_50_ values from the U.S. EPA ECOTOX database, reporting values for Rainbow Trout (Oncorhynchus mykiss) 96-h acute toxicity tests. Values reported as a range of the minimum to maximum LC_50_ values. NA = not available, no data for penconazole fit our criteria.

Chemical	5 PSU LC_10_	15 PSU LC_10_	5 PSU LC_50_	15 PSU LC_50_	FW LC_50_ *
Bifenthrin	0.0000227	0.00000533	0.000160	0.000120	0.00015–0.0147
Chlorpyrifos	0.000137	0.0000393	0.00803	0.00240	<0.001–0.301
Dicloran	0.000288	0.0000704	0.00617	0.00224	0.56–1.6
Myclobutanil	0.00186	0.00430	3.76	1.55	4.2–5.27
Paraquat	0.0547	0.0245	37.50	18.14	15.0–38.68
Penconazole	0.000235	0.0000244	0.107	0.0202	NA
Triadimefon	0.118	0.0123	2.74 ^a^	0.218 ^a^	4.1–15.0

## Data Availability

All data generated in this study and R code used for statistical analysis has been made publicly available and can be found at https://doi.org/10.5281/zenodo.4735670.

## Supplementary Materials

The following are available online at https://www.mdpi.com/article/10.3390/toxics9050114/s1, Table S1: Water quality data for all bioassays including pre- and post-water solutions. ND = Not Detected. All values reported as mean ± standard error [76]. Table S2: Pseudo R2 value used to quantify curve goodness of fit calculated from Nagelkerken’s (1991) pseudo-R2 formula. Video S1: Glass well plate development (double click to play).
