# Supplementary material for: Salinity Alters Toxicity of Commonly Used Pesticides in a Model Euryhaline Fish Species (Menidia beryllina)
Source: Toxics. 2021 May 20;9(5):114. doi: 10.3390/toxics9050114 (PMC8161390; doi:10.3390/toxics9050114)
Supplement: Supplementary file 1 [file toxics-09-00114-s001.zip › toxics-1186974-supplementary 5.22.pdf]

Supplementary Material: Salinity Alters Toxicity of Commonly Used Pesticides in a Model Euryhaline Fish Species (*Menidia beryllina*)

Sara J. Hutton, Scott J. St. Romain, Emily I. Pedersen, Samreen Siddiqui, Patrick E. Chappell, J. Wilson White, Kevin L. Armbrust and Susanne M. Brander

Table S1. Water quality data for all bioassays including pre- and post-water solutions. ND = Not Detected. All values reported as mean ± standard error.

Water Quality	pH	Dissolved Oxygen (mg/L)	Salinity (ppt)	Temperature °C	Ammonia (ppm)
Pre 5ppt	8.51 ± 0.033	7.30 ± 0.055	5.00 ± 0.0023	23.04 ± 0.055	ND
Post 5ppt	7.90 ± 0.037	6.79 ± 0.063	6.10 ± 0.041	22.94 ± 0.053	0.041 ± 0.012
Pre 15ppt	8.37 ± 0.019	7.026 ± 0.053	14.99 ± 0.0025	23.01 ± 0.060	ND
Post 15ppt	7.77 ± 0.030	6.48 ± 0.061	16.86 ± 0.068	22.96 ± 0.050	0.041 ± 0.012

Table S2. Pseudo R2 value used to quantify curve goodness of fit calculated from Nagelkerken's (1991) pseudo-R2 formula.

Chemical	5PSU Pseudo R^2	15PSU Pseudo R^2
Bifenthrin	0.822	0.728
Chlorpyrifos	0.786	0.776
Dicloran	0.693	0.679
Myclobutanil	0.780	0.795
Paraquat	0.753	0.744
Penconazole	0.405	0.548
Triadimefon	0.806	0.824

Supplemental Information 1: Glass Well Plate Development

Twelve-well glass plates were designed and developed for the purpose of conducting high-throughput testing. To design the glass wells the top lip of 10 mL beakers were cut using a 0.75 HP 7 in. 4.8 Amp Tabletop Tile Saw (RYOBI, Anderson, SC) so the beakers were 1 inch high and fit closely together. The tops of the beakers were manually sanded using sandpaper by wetting the sandpaper with R.O. and sanding in a circle until the tops were smooth. The base of the twelve-well plate was printed at Oregon State University on a Prusa i3 Mk2S 3-D printer with a MultiMaterial (MM) upgrade using clear and transparent polylactide polymer (PLA) and designed to match the dimensions of a standard twelve-well plate. Each plate holds twelve 10 mL beakers (4 × 3). The wells can hold up to 10 mL of volume and were filled with 8mL of exposure solution in the presented study. The well plates allow for high throughput testing using fewer organisms, which aligns well with the goals of TOX21 [1]. A visual description is provided in Supplemental Video 1.

Video S1. Glass well plate development. Available online at https://www.youtube.com/watch?v=F-eF1tig2zM.

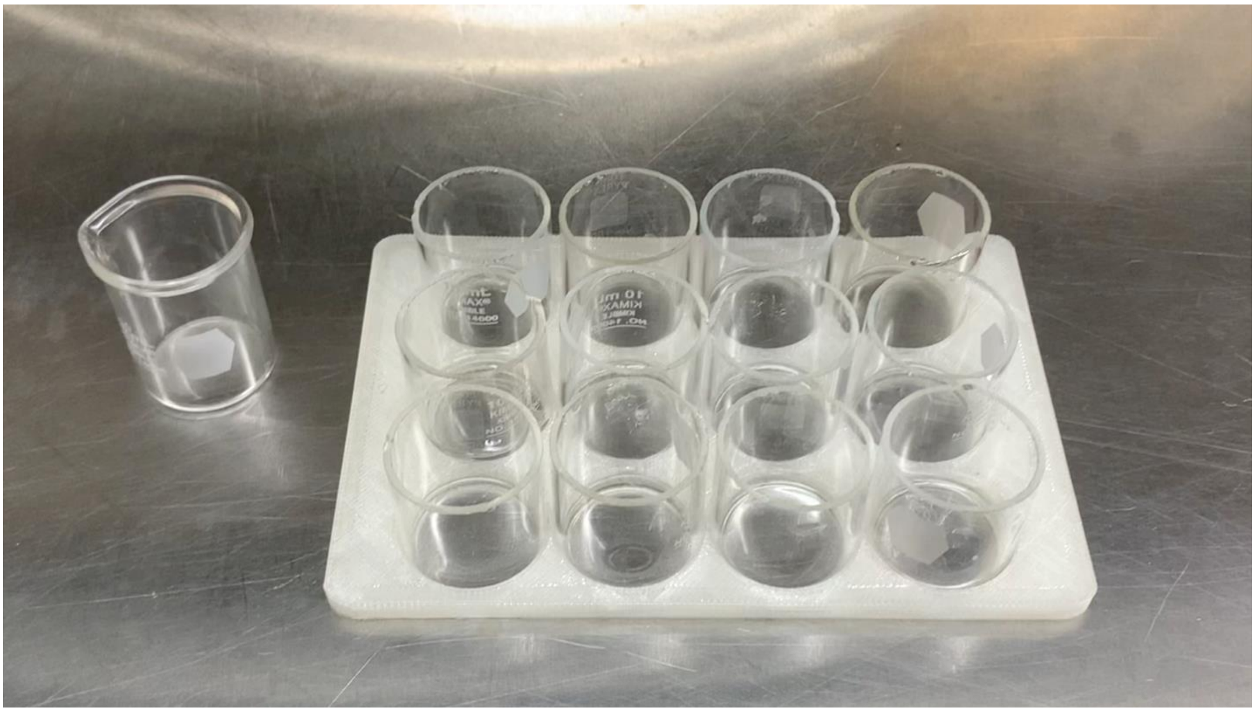

References

1. National Research Council, Toxicity Testing in the 21st Century: A Vision and a Strategy, 2007, Washington, DC: The National Academies Press, doi:10.17226/11970.
